# Ground-Dwelling Arthropod Communities of a Sky Island Mountain Range in Southeastern Arizona, USA: Obtaining a Baseline for Assessing the Effects of Climate Change

**DOI:** 10.1371/journal.pone.0135210

**Published:** 2015-09-02

**Authors:** Wallace M. Meyer, Jeffrey A. Eble, Kimberly Franklin, Reilly B. McManus, Sandra L. Brantley, Jeff Henkel, Paul E. Marek, W. Eugene Hall, Carl A. Olson, Ryan McInroy, Emmanuel M. Bernal Loaiza, Richard C. Brusca, Wendy Moore

**Affiliations:** 1 Department of Entomology, University of Arizona, Tucson, Arizona, United States of America; 2 Museum of Southwestern Biology, University of New Mexico, Albuquerque, New Mexico, United States of America; 3 Department of Ecology and Evolutionary Biology, University of Arizona, Tucson, Arizona, United States of America; 4 University of Sonora, Hermosillo, Sonora, Mexico; 5 Arizona-Sonora Desert Museum, Tucson, Arizona, United States of America; Landcare Research, NEW ZEALAND

## Abstract

The few studies that have addressed past effects of climate change on species distributions have mostly focused on plants due to the rarity of historical faunal baselines. However, hyperdiverse groups like Arthropoda are vital to monitor in order to understand climate change impacts on biodiversity. This is the first investigation of ground-dwelling arthropod (GDA) assemblages along the full elevation gradient of a mountain range in the Madrean Sky Island Region, establishing a baseline for monitoring future changes in GDA biodiversity. To determine how GDA assemblages relate to elevation, season, abiotic variables, and corresponding biomes, GDA were collected for two weeks in both spring (May) and summer (September) 2011 in the Santa Catalina Mountains, Arizona, using pitfall traps at 66 sites in six distinct upland (non-riparian/non-wet canyon) biomes. Four arthropod taxa: (1) beetles (Coleoptera), (2) spiders (Araneae), (3) grasshoppers and crickets (Orthoptera), and (4) millipedes and centipedes (Myriapoda) were assessed together and separately to determine if there are similar patterns across taxonomic groups. We collected 335 species of GDA: 192/3793 (species/specimens) Coleoptera, 102/1329 Araneae, 25/523 Orthoptera, and 16/697 Myriapoda. GDA assemblages differed among all biomes and between seasons. Fifty-three percent (178 species) and 76% (254 species) of all GDA species were found in only one biome and during only one season, respectively. While composition of arthropod assemblages is tied to biome and season, individual groups do not show fully concordant patterns. Seventeen percent of the GDA species occurred only in the two highest-elevation biomes (Pine and Mixed Conifer Forests). Because these high elevation biomes are most threatened by climate change and they harbor a large percentage of unique arthropod species (11–25% depending on taxon), significant loss in arthropod diversity is likely in the Santa Catalina Mountains and other isolated mountain ranges in the Southwestern US.

## Introduction

Rising concentrations of greenhouse gasses in the atmosphere are leading to increasing global temperatures and changes in the hydrological cycle [1]. While such changes are impacting the distribution of biota across the planet [2, 3], research has increasingly focused on mountain environments. High elevation montane species, which are often endemic to a single mountain range, are particularly vulnerable to climate change [4–7] because populations tend to be small, isolated from other source populations, climatically restricted, and unable to move to higher elevations upon reaching the summit of the mountain [8–10]. As a result, these species and populations are especially prone to extirpation or extinction. Thus, elevation gradients on mountains have great potential to enhance our understanding of how climate change will impact biological communities [11, 12]. Elevation gradients are favored over latitudinal gradients since parameters can be measured on a smaller spatial scale, reducing the influence of confounding factors such as large differences in weather patterns and species pools [13, 14]. Therefore, documenting the diversity and distribution of montane species, which establishes a baseline for future comparisons and studies that track how species respond to climate change, is vital to making informed decisions concerning the preservation and management of biodiversity.

Although 85% of all animal species are arthropods (insects, spiders, millipedes, etc.; [15]), studies examining compositional changes and distributional shifts of ground-dwelling (soil/leaf litter) arthropods (GDA) are less common than those examining shifts in plants (see references in [7]), vertebrates, and other arthropod taxa (e.g., butterflies and moths) [3]. In the Southwest, surveys of GDA or arthropods in general are rare. For example, Richmond and O’Keefe [16] highlight that their study is the only comprehensive study in North American of high-elevation (e.g., mountain) arthropod assemblages. In most systems, GDA are poorly known because they are extremely diverse, live in cryptic habitats where observation is difficult, and relatively few taxonomists have specialized on these groups [17]. The lack of information on GDA is disconcerting because they are known to play significant roles in the functioning of healthy ecosystems [18–23]. For example, GDA have been found to significantly influence rates of decomposition in areas where temperature (extremely low or high) and humidity (low) do not constrain arthropod activity [21–23]. Because the annual input of CO_2_ into the atmosphere through decomposition of organic carbon is nearly 10 times that of annual fossil fuel emissions [24], understanding how soil arthropod assemblages will respond to climate change and other anthropogenic perturbations is crucial if we are to understand ecosystem responses and feedbacks [25].

The Santa Catalina Mountains (hereafter, “Catalina Mountains”) in southeastern Arizona offer a rare opportunity to collect species from multiple biomes, test the factors that influence species diversity and composition, and examine the effects of climate change on multiple biomes over relatively short distances [26]. Here, many of the world’s biomes, or major plant communities, occur in climatically structured elevation zones [26–28]. Biomes in the Catalina Mountains include Desertscrub, Desert Grassland, and Oak-Grassland at lower elevations, and Oak Woodland, Pine-Oak Woodland, Chaparral, Pine Forest, and Mixed Conifer Forest at higher elevations. Patterns along elevation gradients in the Catalina Mountains are in many ways analogous to North American latitudinal climatic gradients found from subtropical latitudes (e.g., northern Mexico) to cold temperate latitudes (e.g., Canada) [26–27]. In addition, studies in the Catalina Mountains reveal that climate warming and changes in precipitation have already impacted the life cycles and distributions of many montane plant species [29–31]. Delays in the occurrence of winter rains have caused many annual plants to shift their germination times later in the year [29], while changes in summer monsoon rain patterns have led to significant increase in the elevation at which some plant species flower [30, 32]. Similarly, by comparing data from plant surveys conducted along our transects in 2011 to those of Whittaker and Niering [33] conducted nearly 50 years earlier, Brusca *et al*. [31] showed that the elevation range of many plants in the Catalina Mountains has shifted upslope. This empirical evidence supports model predictions of how plant communities in the Desert Southwest will respond to continued climate warming [1,34,35], and that the area occupied by higher elevation montane forests is decreasing and will likely continue to decrease in the future. This, combined with projections that temperatures in the Southwest will increase by an additional 3–6°C by the end of this century [34, 36–38], make the Catalina Mountains an ideal natural laboratory to investigate the effects of climate change [26].

In this paper, we examine how GDA assemblages, groups of statistically recurring species, are structured in the Catalina Mountains along two elevation gradients. Most previous studies examining elevational distributions of arthropod species have focused on changes within a single taxon, although it has been unclear if such patterns can be generalized across taxa [39, 40]. In addition, many studies have focused on discerning only patterns of species richness, which have yielded mixed results depending on the taxon investigated and the mountain surveyed [14]. Focusing on changes in species richness ignores that elevation also influences the composition of arthropod species assemblages [41–42]. Here, we analyze species composition and abundance of four evolutionarily distinct arthropod lineages: (1) beetles (Coleoptera), (2) spiders (Araneae), (3) grasshoppers and crickets (Orthoptera), and (4) millipedes and centipedes (Myriapoda). Our analyses incorporate species-level identifications and abundance data to determine if there are concordant patterns across taxa (e.g., do community patterns among different ground-dwelling arthropod groups co-vary?) [43]. For each arthropod group (and for combined GDA) we address the following questions: (1) How are GDA assemblages distributed in relation to biomes (e.g., do arthropod assemblages mirror plant communities)? (2) How do assemblages vary between spring/May (pre-monsoon) and summer/September (monsoon season)? (3) What proportion of the species is restricted to a single biome? And, (4) What proportion of the species is only found in high elevation biomes where populations have a higher probability of extinction due to climate change? This is the first detailed investigation of GDA distributions in the Catalina Mountains, or any Sky Island range in the Southwest. Results provide an understanding of current GDA diversity and distribution patterns in the Catalina Mountains, offer a baseline for future comparisons, and demonstrate the risk climate change poses to high-elevation montane communities.

## Materials and Methods

### Study area

Surveys were conducted in the Catalina Mountains, situated 140 km (85 miles) north of the U.S.-Mexico border near Tucson, Arizona, USA (See maps in [26, 28] and S1 File. map of study area). In the Catalina Mountains, many of the world’s biomes (classified by predominant vegetation) occur in climatically structured elevation zones [26]. The Catalinas are one of about 65 mountain ranges in Arizona, New Mexico and Sonora (Mexico) known as the Madrean Sky Islands. These ranges form “stepping stones” across the Cordilleran Gap, between the Rocky Mountains/Colorado Plateau and the Sierra Madre Occidental of Mexico [26, 28]. There is a winter rainy season December through February, and a summer rainy season July through September that is part of the North American Monsoon system [44]. Summer (monsoon) rains account for approximately half the annual precipitation.

### Sampling design

To assess the diversity and distribution of GDA, 66 sampling sites were identified in recognizable biomes along the elevation gradients on the southern and northern sides of the Catalina Mountains. Sites were located along the Mt. Lemmon/Catalina Highway (south side), the Control Road (north side), and on the two highest peaks, Mt. Lemmon (2791 m) and Mt. Bigelow (2591 m). We used Niering and Lowe’s [45] classification system for biome designations, as modified by Moore *et al*. [26] based on an analysis of plant species at each site: Desertscrub, Desert Grassland, Grazing-Disturbed Desert Grassland, Oak-Grassland, Oak Woodland, Pine-Oak Woodland, Chaparral, Pine Forest, and Mixed Conifer Forest (see Moore et al., [26] and Brusca and Moore [28] for detailed descriptions of plant communities and exact locations and elevations of each sampling site). For analyses in this study we combined all three grassland biomes. We also combined Oak Woodland sites with Pine-Oak Woodland sites since pure Oak Woodland is found only in a narrow elevational zone primarily on the north side of the Catalina Mountains and represents the lower elevational boundary of the extensive Pine-Oak Woodland, often considered an ecotone [25]. On the south side of the range, we established 5 sites in Desertscrub (1045–1172 m), 6 in Oak-Grassland (1384–1433 m), 7 in Pine-Oak Woodland (1803–2422 m), 2 in Chaparral (1923–2052 m), and 8 in Pine Forest (2224–2463 m). On the north side of the range we established 7 sites in grazing-disturbed Desert Grassland (historically grazed areas; 1323–1451 m), 6 in relatively undisturbed Desert Grassland (1330–1645 m), 2 in Oak Woodland (1939–2000 m), 5 in Pine-Oak Woodland (2032–2149 m), 5 in Chaparral (1845–1971 m), and 4 in Pine Forest (2218–2305 m). Ten transects in Mixed Conifer Forest were established near the peaks of Mt. Lemmon and Mt. Bigelow at 2442 to 2777 m elevation.

In addition to categorizing sites according to biome, we also categorized each site according to three environmental variables: elevation, ground temperature, and ground-level humidity (~ 2 cm above the soil/litter surface). We report both temperature and ground-level humidity because these two factors contribute to understanding desiccation stress. As humidity increases, desiccation stress declines. Environments with higher temperatures are more desiccating than low temperature environments with an identical humidity. Ground temperature and humidity were measured using Log-Tag HAXO-8 temperature and humidity recorders. Each Log-Tag recorder was covered with a plastic shield and placed 2 cm from the center of each of the 66 site transects on May 6, 2011. Data were recorded every 30 min through the entire sampling period.

We sampled arthropods for two weeks in the spring (pre-monsoon; May 5–19) and two weeks in the summer (monsoon season; September 1–15) of 2011. At each sampling site, we set 10 pitfall traps arranged 10 m apart along a 100 m transect line. Using the trap design employed by Higgins [46], pitfall traps were constructed so they could remain in the field for two weeks between charging of a trap and collection of specimens. Each pitfall trap consisted of a heavy Pyrex glass “test tube” (3.2 cm diameter, 25 cm deep) inserted in a PVC sleeve (3.8 cm in diameter, 28 cm long) buried in the ground. The opening of each trap was flush with the soil surface. When charged, test tubes were half filled with ~ 75 ml of propylene glycol, which is non-toxic and is an excellent short-term preservative for arthropods. Each pitfall trap was covered with a PVC rain shield with holes ~ 3–4 cm above the ground to allow arthropods access while restricting access to most vertebrates. Between sampling periods, PVC sleeves were capped to prevent them from filling with soil or inadvertently capturing vertebrates. Strictly speaking, pitfall traps capture activity abundance, i.e., the relative abundance of those species active during the sampling period.

Contents collected in each pitfall trap were transferred from propylene glycol to 80% ethanol and stored in a –20°C freezer prior to processing. Arthropods were sorted and four taxonomic groups were analyzed: (1) beetles (Coleoptera), (2) spiders (Araneae), (3) grasshoppers and crickets (Orthoptera), and (4) millipedes and centipedes (Myriapoda). Members of all four groups are distributed across the entire elevation gradient. The Moore laboratory (Univ. Arizona) has the taxonomic expertise and/or collaborators specializing on these groups. All individuals were sorted to morphospecies. For Coleoptera, Araneae, and Orthoptera only adult specimens were identified. All specimens were curated, labeled and deposited in the University of Arizona Insect Collection (http://www.uainsectcollection.com/?q=ento/UAIC/).

### Analyses

To compare abiotic characteristics among sites and examine differences between the spring and summer sampling periods, mean daily temperatures and relative humidity averages recorded during each collection period were plotted against elevation. To test if average daily temperature and humidity differed between the two sampling periods, we used a paired t-test that controls for among-site differences. We used a simple linear regression to analyze how temperature and humidity conditions changed along the elevation gradients and to confirm that these two characteristics are related to changes in elevation.

To investigate differences in GDA composition among biomes and between seasons, we used permutation-based hypothesis testing (ANOSIM analyses) implemented in PRIMER 6.1.15 [47]. Five separate analyses were conducted: one used the combined data from all 4 taxa, and four examined each GDA group separately (S2 File; site by species matrix). We accounted for multiple hypothesis tests by adjusting significant values with the conservative Bonferroni procedure [48]. Prior to all ANOSIM analyses, we constructed a site-by-species matrix using the average number of individuals per species collected per pitfall trap at each site. This was done to standardize abundance measures among sites to account for lost or damaged traps, which was primarily an issue in low elevation biomes during the summer (monsoon) collection period. Abundance data were square root transformed to reduce the influence of extremely abundant taxa. Following standardization and transformation, similarity matrices were constructed using the Bray-Curtis similarity coefficient. For each ANOSIM analysis, we report both the ANOSIM test statistic (R-values) and the permutation based P-values. The R-statistics are the average rank dissimilarities among and within groups, scaled so that R-values vary between roughly 0 and 1 (there may be some negative values); a value of 0 indicates that there are no differences among treatments, and a value of 1 indicates that all dissimilarities between samples in different treatments are larger than the average dissimilarity among samples within each treatment. The P-values test whether R-statistics differ significantly from zero (i.e., that there are significant differences among treatments).

To test for significant differences in arthropod assemblages among biomes and between sampling periods, we used a two-way crossed ANOSIM (9,999 permutations) using biome (Desertscrub, Grassland, Chaparral, Oak, Pine and Mixed Conifer) and sampling period (spring [pre-monsoon] and summer [monsoon]) as factors. We chose a two-way crossed ANOSIM which treats the season factor as an independent fixed effect, because it is a conservative (i.e., less statistical power to reject the null) alternative to using a Repeated Measures PERMANOVA which would take into account any positive correlation associated with sites. This allowed us to test for differences in arthropod assemblages among biomes controlling for sampling period and to test for differences among seasons controlling for differences among biomes. Pairwise differences were only examined following a significant ANOSIM test with significance values adjusted for multiple testing (α = 0.01). Multi-dimensional scaling (MDS) plots were generated following significant ANOSIM tests to visualize differences in arthropod assemblage among biomes and seasons. If GDA assemblages were found to significantly differ among biomes, one-way ANOSIMs were run to test differences in arthropod assemblages among adjacent biomes within each season (10 comparisons; α = 0.005). If arthropod assemblages were found to significantly differ among sampling periods, one-way ANOSIMs were run to test differences in arthropod assemblages between each sampling period in each biome (6 comparisons; α = 0.0083). Similarity percentage analyses (SIMPERs) were run using only the combined four-taxa matrix following significant pairwise comparison tests to determine the relative contribution of the various taxa to these differences (e.g., which species are driving the differences in the arthropod communities).

In addition to ANOSIM and SIMPER tests, we also compared the proportion of species that were exclusively found in one biome and the proportion of species only found in the two highest biomes (Pine and Mixed Conifer) to better understand community composition along the elevation gradient.

## Results

**Fig 1:**
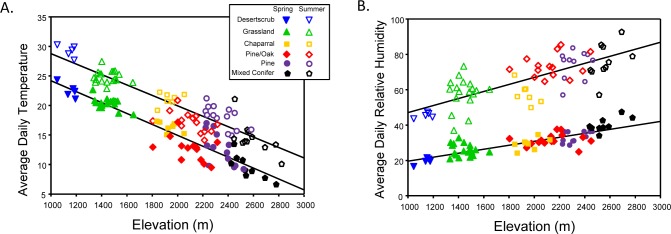
Ground temperature and relative humidity along the elevation gradient. Average temperatures (A) and relative humidity (B) measured during the spring (6-May-2011 to 19-May-2011) and late-summer (1-September-2011 to 15-September-2011) seasonal sampling periods.

Ground temperature and humidity were both significantly related to elevation during both sampling periods (spring temperature: slope -0.009, r^2^ = 0.874, P < 0.0001; summer temperature: slope -0.008, r^2^ = 0.847, P < 0.0001; spring humidity: slope 0.011, r^2^ = 0.771, P = 0.0001; summer humidity: slope 0.020, r^2^ = 0.5597, P < 0.0001), and both average daily temperature (t = 25.32, p < 0.0001) and humidity (t = 31.89, p = < 0.0001) were significantly higher in summer (Fig 1).

All individuals of Coleoptera, Araneae and Myriapoda were sorted to species (identified to a species name, or to a morphospecies code when species-level identification was not possible). For Orthoptera, taxonomic uncertainty (e.g., Jerusalem crickets, genus *Stenopelmatus*) and absence of key characteristics (e.g., calls are required for field cricket identification in the genus *Gryllus*), meant that individuals in some taxa were sorted to genus and therefore these few taxa might represent multiple species. However, in the latter case, to minimize using multiple terminologies, we still refer to these identified groups as “species,” since the majority of the taxa do represent species-level distinction.

**Fig 2:**
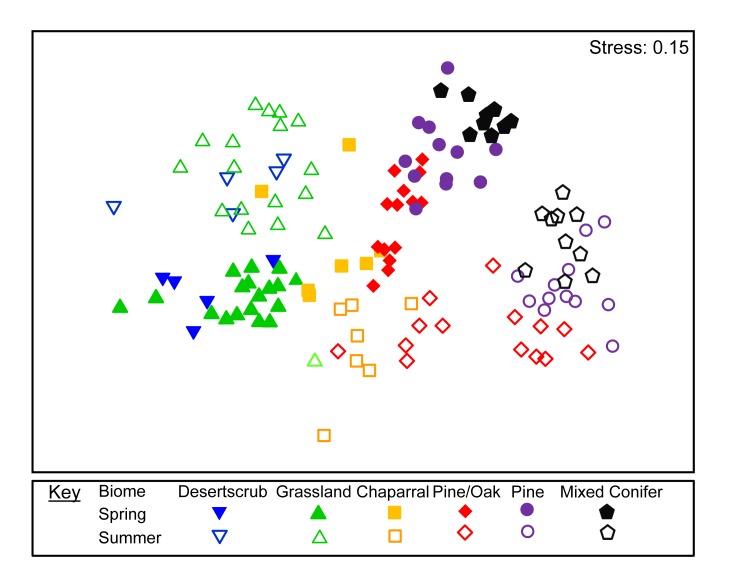
MDS ordinations for all four arthropod taxa. MDS ordination of sites according to the composition and abundance (square root transformed) using the combined data set of all four arthropod taxa (Coleoptera, Araneae, Orthoptera and Myriapoda). Similarity determined using the Bray-Curtis similarity coefficient. Sites that are closer together are more similar in arthropod composition.

**Fig 3:**
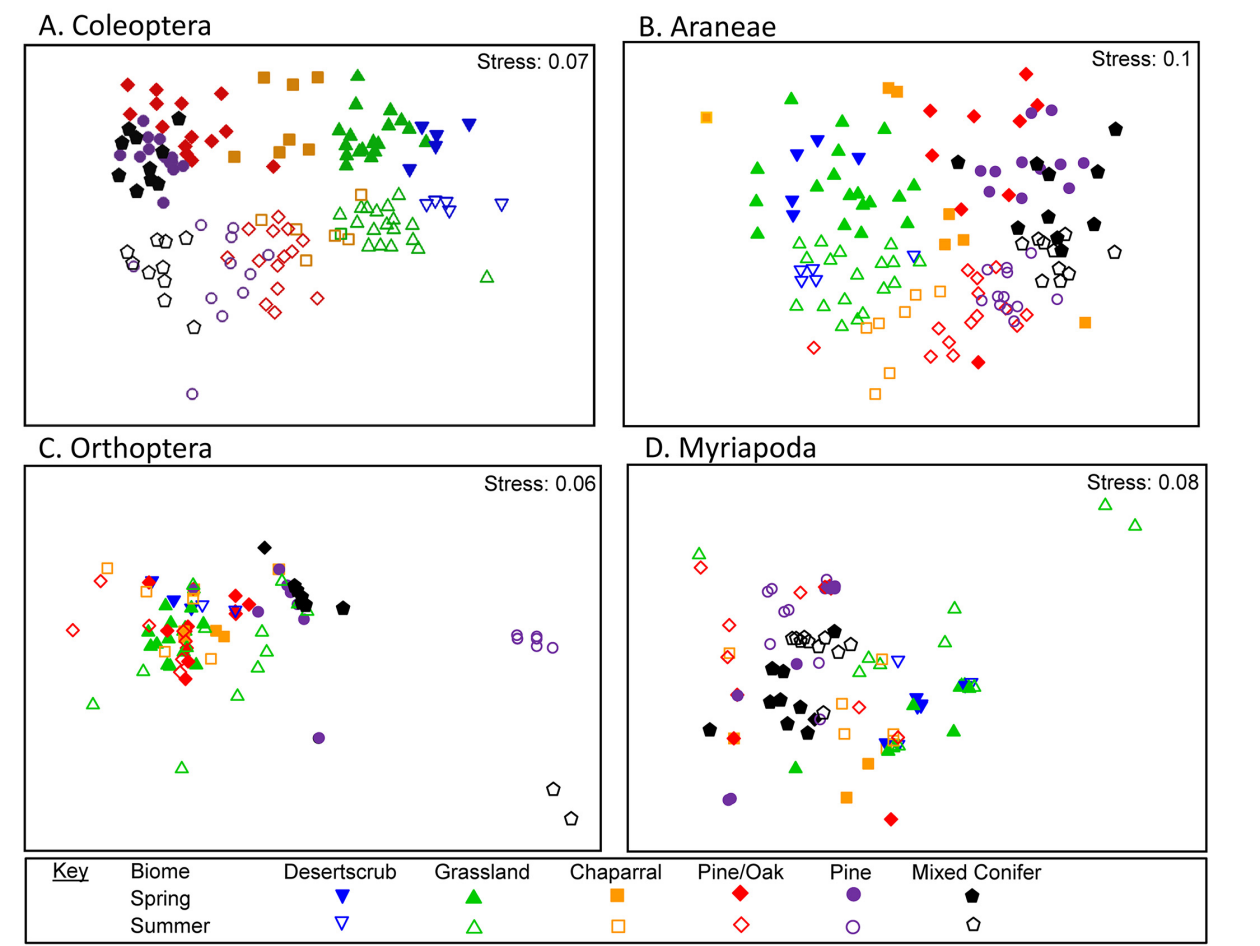
MDS ordinations of sites according to: Coleoptera (A), Araneae (B), Orthoptera (C) and Myriapoda (D) composition and abundance (square root transformed). Similarity determined using the Bray-Curtis similarity coefficient. Sites that are closer together are more similar in arthropod composition.

We collected and curated 335 species (and 6342 individuals); 192/3793 (species/specimens) Coleoptera, 102/1329 Araneae, 25/523 Orthoptera and 16/697 Myriapoda (S3 File. species lists indicating plant biome and season collected). ANOSIM analyses revealed significant differences in species composition between biomes (R = 0.77, P = 0.0001; Fig 2; Table 1) and between seasons (R = 0.86, P = 0.0001; Fig 2; Table 2) when data from all four taxa were analyzed together. When each GDA group was analyzed separately, we again found significant differences between biomes (Coleoptera: R = 0.67, P = 0.0001; Araneae: R = 0.57, P = 0.0001; Orthoptera: R = 0.42, P = 0.001; Myriapoda: R = 0.36, P = 0.0001) and between seasons (Coleoptera: R = 0.79, P = 0.0001; Araneae: R = 0.54, P = 0.001; Orthoptera: R = 0.37, P = 0.0001; Myriapoda: R = 0.17, P = 0.0005) (Fig 3, Tables 3 and 4). While significant differences were observed between all adjacent biomes when we combined the four taxa, that pattern did not hold when each taxon was analyzed independently (Tables 1 and 3). SIMPER analyses revealed that significant differences in assemblages among neighboring biomes, and between seasons, are driven both by differences in species composition (species turnover) and by large shifts in abundance of species (Tables 2 and 4).

**Table 1:** Results from pairwise ANOSIM tests comparing differences in arthropod assemblages among adjacent biomes in each season (spring/May and summer/September). Significant differences following Bonferroni adjustment (α = 0.005) are asterisked.

Season	Pairwise Comparisons	All Taxa		Coleoptera			Araneae		Orthoptera		Myriapoda	
		R	P	R	P	R		P	R	P	R	P
May	Desert scrub vs. Grassland	0.50	0.0003*	0.63	0.0005*	0.07		0.2330	0.30	0.0530	-0.14	0.9800
	Grassland vs. Chaparral	0.63	0.0001*	0.50	0.0004*	0.53		0.0001*	0.33	0.0090	0.18	0.0470
	Chaparral vs. Pine/Oak	0.55	0.0001*	0.34	0.0020*	0.62		0.0001*	-0.07	0.7700	0.02	0.3910
	Pine/Oak vs. Pine	0.34	0.0001*	0.27	0.0002*	0.08		0.0600	0.34	0.0010*	0.11	0.0910
	Pine vs. Mixed Conifer	0.59	0.0001*	0.32	0.0020*	0.61		0.0001*	0.17	0.0120	0.29	0.0060
September	Desert scrub vs. Grassland	0.43	0.0010*	0.49	0.0004*	0.11		0.1360	-0.22	0.9580	-0.14	0.8560
	Grassland vs. Chaparral	0.72	0.0001*	0.78	0.0001*	0.32		0.0005*	0.08	0.1700	0.11	0.1190
	Chaparral vs. Pine/Oak	0.47	0.0001*	0.54	0.0004*	0.30		0.0010*	-0.07	0.8460	0.01	0.3560
	Pine/Oak vs. Pine	0.27	0.0004*	0.10	0.0500	0.16		0.0070	0.78	0.0003*	0.10	0.1240
	Pine vs. Mixed Conifer	0.47	0.0002*	0.23	0.0050	0.00		0.4500	1.00	0.0280^ϯ^	0.45	0.0004*

Ϯ Lowest possible p-value due to limited possible permutations, but not significant following α correction.

**Table 2:** Results from SIMPER analyses, listing the six most important species according to its contribution to the dissimilarity between arthropod assemblages in adjacent biomes: DS = Desertscrub, GL = Grassland, CH = Chaparral, and PO = Pine Oak, P = Pine, MC = Mixed Conifer. Capital letters preceding species names indicate to which major arthropod group the species belongs (C-Coleoptera, A- Araneae, O-Orthoptera, M-Myriapoda).

*Spring Comparisons*	No.		Contribution	*Summer Comparisons*	No.		Contribution
	Per		%		Per		%
Species	Trap		Dissimilarity	Species	Trap		Dissimilarity
**DS vs GL**	**DS**	**GL**		**DS vs GL**	**DS**	**GL**	
C *Triorophus laevis*	4.34	0.00	11.61	C *Eusattus reticulatus*	0.08	1.25	6.97
C *Steriphanus subopacus*	0.72	0.00	5.43	C *Microlestes linearis*	0.86	0.43	5.75
A *Syspira longipes*	0.48	0.15	4.04	C *Pasimachus californicus*	0.00	0.54	5.49
C *Cryptoglossa variolosa*	0.38	0.00	4.02	C *Discoderus robustus*	0.53	0.39	4.92
C *Agroporis costipennis*	0.14	0.85	3.98	A *Ceraticelus* nr. *formosus*	0.26	0.04	3.63
O *Gryllus* spp.	0.10	0.48	3.25	A *Steatoda variata*	0.28	0.03	3.31
**GL vs CH**	**GL**	**CH**		**GL vs CH**	**GL**	**CH**	
C *Agroporis costipennis*	0.85	0.16	5.71	C *Eusattus reticulatus*	1.25	0.07	6.20
C *Eleodes* spp.	0.02	0.31	3.83	C *Onthophagus* sp.	0.01	1.21	5.18
O *Gryllus* spp.	0.48	0.26	3.79	C *Pasimachus californicus*	0.54	0.00	4.77
A *Callilepis gertschi*	0.19	0.31	3.57	O *Gryllus* spp.	0.29	0.43	3.98
A *Zelotes monachus*	0.32	0.00	3.39	C *Opatrinae* spp.	0.16	0.40	3.54
A *Drassyllus insularis*	0.21	0.00	2.92	C *Microlestes linearis*	0.43	0.00	3.35
**CH vs PO**	**CH**	**PO**		**CH vs PO**	**CH**	**PO**	
C *Serica* sp.	0.05	0.76	7.01	C *Euparea* sp.1	0.03	1.55	7.20
A *Callilepis eremella*	0.00	0.29	5.63	C *Onthophagus* sp.	1.21	0.07	6.55
A *Callilepis gertschi*	0.31	0.00	4.91	O *Gryllus* spp.	0.43	0.46	5.22
C *Eleodes* spp.	0.31	0.34	4.81	C *Opatrinae* spp.	0.40	0.02	4.97
O *Gryllus* spp.	0.26	0.36	4.45	C *Stelidota geminata*	0.01	0.46	4.61
A *Alopecosa kochi*	0.00	0.20	3.64	C *Synuchus dubius*	0.00	0.39	4.45
**PO vs P**	**PO**	**P**		**PO vs P**	**PO**	**P**	
C *Serica* sp.	0.76	0.72	8.35	C *Euparea* sp.1	1.55	3.59	12.64
O *Gryllus* spp.	0.36	0.02	5.56	C *Stelidota geminata*	0.46	0.63	5.75
A *Alopecosa kochi*	0.20	0.46	5.15	O *Gryllus* spp.	0.46	0.00	4.55
C *Eleodes* spp.	0.34	0.02	4.42	A *Pardosa montgomeryi*	0.09	0.36	4.48
O *Ceuthophilus* spp.	0.07	0.25	4.00	A *Xysticus montanensis*	0.00	0.33	4.41
A *Callilepis eremella*	0.29	0.22	3.88	C *Synuchus dubius*	0.39	0.36	4.30
**P vs MC**	**P**	**MC**		**P vs MC**	**P**	**MC**	
A *Callobius arizonicus*	0.04	0.57	7.02	M *Aniulus* sp.1	0.56	3.52	10.85
C *Serica* sp.	0.72	0.00	5.57	C *Euparea* sp.1	3.59	1.74	9.10
O *Ceutophilus* spp.	0.25	0.32	4.64	C *Synuchus dubius*	0.36	0.97	4.34
C *Cryptophagus* sp1	0.06	0.24	4.53	M Lithobiid sp.3	0.14	0.59	3.78
A *Callilepis eremella*	0.22	0.00	4.44	C *Stelidota geminata*	0.63	0.01	3.77
C *Omaliinae* sp1	0.00	0.54	4.14	A *Pardosa montgomeryi*	0.36	0.04	3.38

**Table 3:** Results for pairwise ANOSIM tests comparing differences in arthropod assemblages between seasons in each biome. Significant differences following Bonferroni adjustment (α = 0.0083) are asterisked.

	All Taxa		Coleoptera		Araneae		Orthoptera		Myriapoda	
Biome	R	P	R	P	R	P	R	P	R	P
Desertscrub	0.86	0.0080*	0.69	0.0080*	0.85	0.0080*	0.11	0.4000	0.18	0.1400
Grassland	0.80	0.0001*	0.71	0.0001*	0.42	0.0001*	0.30	0.0003*	0.11	0.0410
Chaparral	0.66	0.0006*	0.55	0.0006*	0.46	0.0006*	0.11	0.1190	0.19	0.0860
Pine/Oak	0.85	0.0001*	0.90	0.0001*	0.57	0.0001*	0.14	0.0650	0.02	0.2690
Pine	0.98	0.0001*	0.66	0.0001*	0.94	0.0001*	0.67	0.0004*	0.00	0.4600
Mixed Conifer	0.99	0.0001*	0.78	0.0001*	0.36	0.0002*	1.00	0.0150 ^ϯ^	0.59	0.0002*

^Ϯ^ Lowest possible p-value due to limited possible permutations, but not significant following correction.

**Table 4:** Results from SIMPER analyses, listing the six most important GDA species according to its contribution to the dissimilarity within each biome during the two collection periods (spring [pre-monsoon], summer [monsoon]). Capital letters preceding species names indicate to which major arthropod group the species belongs (C-Coleoptera, A- Araneae, O-Orthoptera, M-Myriapoda).

Biome/ Species	No. Per Trap		% Contribution
	Spring	Summer	Dissimilarity
**Desertscrub**			
C *Triorophus laevis*	4.34	0.00	11.97
C *Steriphanus subopacus*	0.72	0.00	5.61
C *Microlestes linearis*	0.12	0.86	4.64
A *Syspira longipes*	0.48	0.04	4.49
A *Callilepis gertschi*	0.30	0.00	4.18
			**30.89**
**Grassland**			
C *Eusattus reticulatus*	0.00	1.25	6.02
C *Agroporis costipennis*	0.85	0.09	4.80
C *Pasimachus californicus*	0.00	0.54	4.42
O *Gryllus* spp.	0.48	0.29	3.98
C *Microlestes linearis*	0.07	0.43	3.09
			**22.31**
**Chaparral**			
C *Onthophagus* sp.	0.00	1.21	6.91
C *Opatrinae* spp.	0.00	0.40	5.33
A *Callilepis gertschi*	0.31	0.00	4.51
C *Eleodes* spp.	0.31	0.03	4.32
O *Gryllus* spp.	0.26	0.43	4.16
			**25.23**
**Pine/Oak**			
C *Serica* sp.	0.76	0.00	7.43
C *Euparea* sp.	0.00	1.55	7.05
O *Gryllus* spp.	0.36	0.46	5.08
A *Callilepis eremella*	0.29	0.00	4.81
C *Stelidota geminata*	0.00	0.46	4.63
			**29.00**
**Pine**			
C *Euparea* sp.	0.00	3.59	9.71
A *Alopecosa kochi*	0.46	0.03	5.76
C *Synuchus dubius*	0.00	0.36	4.64
C *Serica* sp.	0.72	0.00	4.21
A *Pardosa montgomeryi*	0.00	0.36	4.09
			**28.41**
**Mixed Conifer**			
M *Aniulus* sp.1	0.10	3.12	8.35
C *Euparea* sp.	0.00	1.74	6.85
C *Synuchus dubius*	0.00	0.97	5.99
M Lithobiid sp.3	0.03	0.59	4.12
O *Ceuthophilus* spp.	0.32	0.00	3.98
			**29.29**

**Fig 4:**
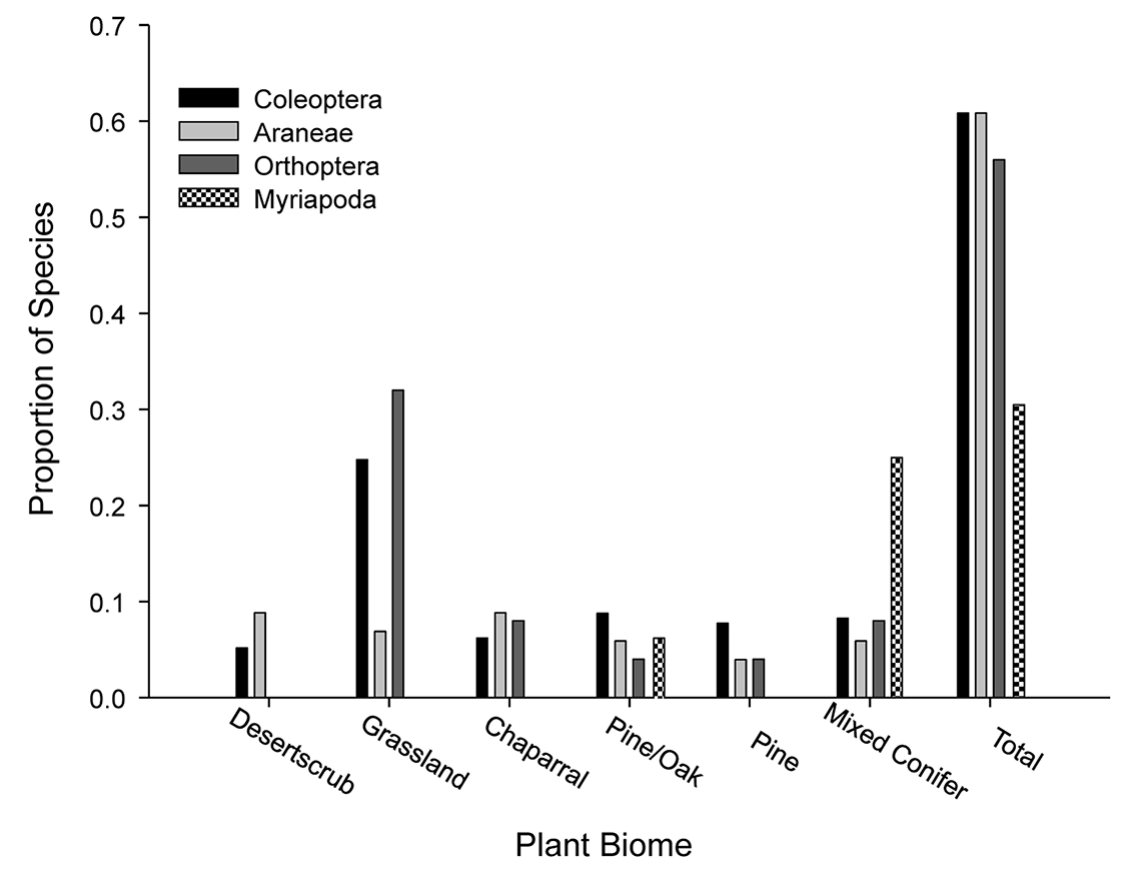
Proportion of species in each taxa found in only one biome. Proportion of species in each taxa (Coleoptera, Araneae, Orthoptera, and Myriapoda) found in only one biome (presented as a proportion of the total richness).

**Fig 5:**
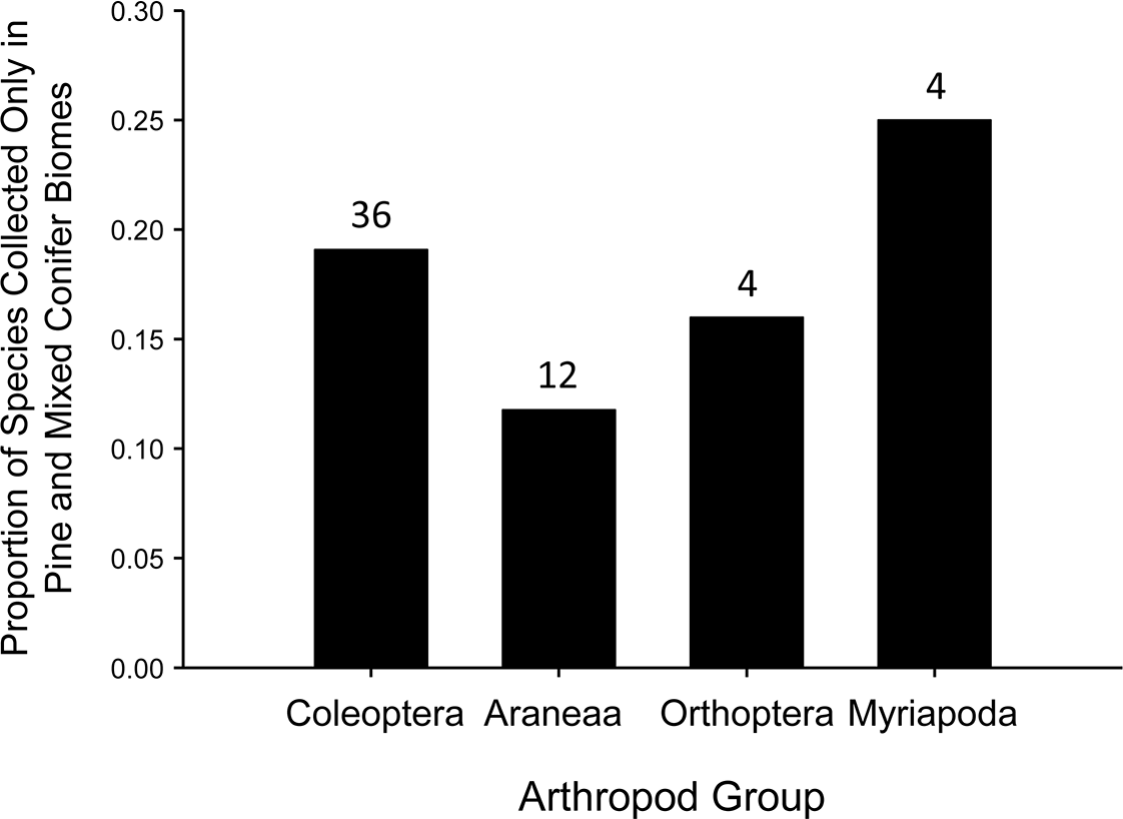
Proportion of species collected that were found only in the two highest elevation biomes. Pine and Mixed Conifer Forests. Numbers above each bar indicate the total number of species.

Fifty-three percent (178 species) of all GDA species collected were found in only one biome, and over 50% of the species of Coleoptera, Araneae, and Orthoptera occurred in only one biome (Fig 4; S3 File). Of the 31% of Myriapoda species collected in one biome, 25% percent were collected in the highest elevation biome, Mixed Conifer Forest. Of all GDA species collected, 56 (17%) species occurred only in the two highest-elevation biomes (Pine and Mixed Conifer Forests). The proportion of species within each of the four taxa analyzed confined to these high elevation biomes varied from 11 to 25% (Fig 5).

Seventy-seven percent (254 species) of all GDA species collected were found only during one season (S3). The percent of species collected in only one season was ≥ 50% for each GDA taxon: 84% Coleoptera (162 species), 69% Araneae (70), 56% Orthoptera (14), and 50% Myriapoda (8). The percentage of species collected in only one season was consistent between seasons for both the Coleoptera (40% in the spring and 45% in the summer) and the Myriapoda (25% spring, 25% summer), but varied with a higher percentage of Araneae (46% spring, 23% summer) and a lower percentage of Orthoptera (4% spring, 52% summer) collected in the spring.

## Discussion

Most investigations of arthropod diversity along elevation gradients have focused on patterns of species richness (see [14, 49]). Such studies most often seek to identify elevations that harbor the highest species diversity [4]. However, this approach ignores that elevation also profoundly influences species composition and abundance [41], and that plant biomes change with elevation. Our study incorporated both species identity and abundance into the analyses, and we found that all biomes along the elevation gradient harbor unique GDA assemblages. This pattern was consistent between seasons despite significant turnover in species within biomes between the spring and summer. Differences in GDA assemblages among biomes were partly driven by widespread species having different abundances in the various biomes in which they are present (e.g., *Agroporis costipennis* is more abundant in the Grassland biome than it is in the two adjacent biomes, Desertscrub and Chaparral, in the spring). However, biomes do harbor different GDA species, and more than 50% of the Coleoptera, Araneae, and Orthoptera species were only collected in one biome. Further research is required to ascertain what ecological and/or physiological factors are correlated with this pattern.

Across the Southwest, average annual temperatures have risen over 0.84°C since 1951 [50], and in Arizona the average annual temperature has increased by 1.4°C since 1976 [51]. Average daily temperatures in the Southwest for the 2001–2010 decade were the highest on record since 1900, and the period since 1950 has been warmer than any period of comparable length in at least 600 years [7]. In Tucson (Arizona), mean annual air temperature increased an average of 0.25°C/decade from 1949 to 2011, and mean annual precipitation declined significantly from 1991 to 2011 [15]. Van Mantgem et al. [52] attribute the rapid increase in tree mortality rates in the western United States to higher ambient temperatures. In New Mexico in the 1950s, there was a large shift (of 2 km or more) in the ecotone between semi-arid ponderosa pine forest and piñon-juniper woodland in less than five years as a result of ponderosa pine mortality associated with severe drought [53]. Substantial support exists for projected temperature increases in the U.S. over the rest of this century, and models agree that increases in summer temperatures will be greatest in the Southwest [51].

Species’ vulnerability to environmental change and the nature of that change varies with elevation [54]. While, lower-elevation arthropod species are threatened by habitat disturbance mediated directly by humans [55] or indirectly through climate change [56], including the establishment and spread of invasive weed species (e.g., buffelgrass, *Pennisetum ciliare* in the Catalina Mountains), species at higher elevations everywhere are threatened by climate change and, potentially, the complete loss of their habitat [10]. In the mountains of southwestern U.S., climate change most threatens the survival of animals (and plants) confined to high elevation biomes [30, 54]. This threat is greatest in Pine and Mixed Conifer Forests because these high elevation communities can be extremely isolated and as a result often contain a high proportion of endemic species or lineages [57]. In the Catalina Mountains, we found that 17% (56) of the GDA species collected were only found in these two highest elevation biomes. This proportion of species is consistent with estimates for reptiles and mammals, which suggest 15 to 25% of those species in Arizona are at risk of extinction or local expatriation due to the loss of high elevation habitats as a result of climate change [54]. Studies examining snails, beetles, harvestmen, and jumping spiders have shown that different Sky Island ranges hold genetically distinct populations and species [58–61]. Because the total area of these high elevation biomes is decreasing [31, 34], significant loss of high elevation species can be expected. The threat is also high in Oak Woodlands and Pine-Oak Woodland biomes, where habitat is predicted to be lost to encroaching Desert Grasslands as climates continue to warm and dry in the Southwest [62]. For example, projected vegetation changes in Saguaro National Park (next to the Santa Catalina Mountains), assuming a temperature rise of 4°C this century, include an expansion of both Desertscrub and Desert Grassland, and decreases in woodland and forest habitats [34].If high elevation biomes in these mountains harbor the same species, but unique populations (e.g., evolutionary significant units), loss of biodiversity might be even more pronounced.

These results underscore the need for more field-based inventories and baselines of arthropod taxa throughout the mountains of the Southwest to predict how arthropod assemblages will respond to climate change. However, significant differences in GDA assemblages between seasons (76% of the species were collected only in one season) indicate that this factor must be accounted for when comparing GDA communities between years and among mountain ranges. Because critical temperature thresholds limit growth, development, survival, reproduction and activity of ectothermic arthropods [63, 64],the shift in composition between seasons may be explained by differences in temperature and humidity and the ability of the species to cope with the corresponding changes in desiccation stress. However, controlling for temperature when making inter-mountain comparisons may be difficult in the Madrean Sky Island Region, defined by Moore *et al*. [26] to include the Pinal Mountains in the north (~ 32.2°N in latitude) and the Sierra Mazatán in the south (~29° in latitude), since temperatures vary considerably along this latitudinal gradient within a season. In addition, limited access to most Madrean Sky Islands makes it logistically difficult to carry out comparative studies among multiple ranges. The same considerations must also be taken into account when making year-to-year considerations, since our results highlight that season influences GDA species turnover. Further, these mountain ranges vary substantially in size, adding area as another confounding variable. Comparisons of only the high elevation biomes (Mixed Conifer and Pine Forests) among ranges, where species are most threatened, may be most important and most feasible. Because these biomes cover less area, sampling strategies can be developed to thoroughly inventory arthropod diversity thereby adding confidence to inter-mountain comparisons and endemic taxa requiring direct conservation efforts can be identified.

Perhaps unsurprisingly, patterns were not concordant among GDA taxa (Coleoptera, Araneae, Orthoptera, and Myriapoda) on either geographical or temporal scales. In our study, Coleoptera assemblages differed in every biome along the elevation gradient in spring, but differences were not observed between adjacent high elevation biomes in summer (i.e., Pine and Mixed Conifer Forests). These high elevation biomes were not similar in the summer because phenological differences resulted in beetle species present in the Pine biome in the spring moving higher into the Mixed Conifer Biome in the fall. Instead, these differences were due to a different assemblage of beetles inhabiting these two biomes in the summer. While the scenario that species may move to higher elevation biomes as temperatures warm seems logical, we see no evidence of this in the Catalinas. Instead, we observed high species turnover between seasons indicating that different species are present/active. Similar to beetles, Araneae assemblages consistently differed between Grassland and Chaparral, and Chaparral and Pine-Oak biomes, but differences between Pine and Mixed Conifer biomes in spring were not found in summer. In contrast, the only adjacent biomes that had different Orthoptera assemblages were the Pine-Oak and Pine biomes in both sampling periods, and Myriapoda assemblages differed between Pine and Mixed Conifer biomes only in the summer. This suggests that identifying selected species (or higher taxa) to serve as indicators or surrogates for other taxonomic groups, or for the entire GDA fauna, may not be possible. In addition, both paleontological and contemporary studies suggest that species assemblages do not respond to climate change as entities [31, 65–67]. Rather, species responses tend to be idiosyncratic, resulting in the development of entirely new assemblages and associations [68, 69]. Variation in response to climate change among species result from differences in specific species characteristics (e.g., ability to physiologically cope with the changing conditions and disperse to favorable areas), and complex interactions among species, notably mismatches between predators and prey [70] and herbivorous insects in their host plants [71]. Combined, these results again underscore the need for field-based inventories and baselines of multiple GDA taxa for future comparisons.

Comprehensive surveys and monitoring protocols are required if we are to compile a relatively complete species inventory of GDA in even one mountain range. Since GDA are notoriously diverse [17], an enormous and resource-intensive effort is often required to obtain a relatively complete inventory using pitfall trapping [72]. While this was not the goal of our study, it is interesting to note that even with the 18,480 trap-days employed in our sampling, we are unable to confidently estimate the species richness of Coleoptera or Araneae for the entire mountain range, or even for plant biomes, despite collecting 192 and 102 species in each of these groups, respectively. Large turnover in GDA species between seasons certainly indicates that increased sampling in all biomes will increase species richness estimates. Still, our surveys captured the most abundant and conspicuous species, and future monitoring efforts will allow us to track changes in these more common species while also capturing more of the rare species in an attempt to inventory the total arthropod diversity.

Our study is the first to document the remarkable diversity of ground-dwelling arthropods in one of the 65 ranges that comprise the Madrean Sky Island Mountain Region, and it highlights the potential for a substantial loss of this diversity under climate change. Low elevation biomes (Desertscrub and Grassland) are predicted to expand in area as climates continue to warm in the Southwest [31, 34]; however, they are subject to other threats, such as invasive exotic weeds (e.g., buffelgrass, *Pennisetum ciliare*). In contrast, high elevation montane woodlands and conifer forests are expected to decrease in size [31, 34]. These higher elevation biomes not only harbor a significant proportion of GDA species (11 to 25% of the species in our study, depending on GDA group), but high elevation GDA biotas in the Madrean Sky Island Region are known to be extremely isolated [57] and likely contain a high proportion of genetically distinct populations and species [58–61]. Many of these previous studies focused on GDA such as jumping spiders [59], harvestmen [60], and ground-dwelling carabid beetles [61]. Because both empirical data and model-based projections suggest that the area of these high elevation biomes is decreasing rapidly [31, 34], irreplaceable loss in GDA diversity in the Santa Catalina Mountains as well as other isolated mountain ranges in the southwestern U.S. is likely to occur.

## Supporting Information

S1 File A file to show location of study sites. Color coding of sites according to plant biomes is consistent with Figs 2 and 3.
(PDF)

S2 File A file that provides the site (rows) by species (columns) matrix for all four arthropod taxa. Categorical and continuous ecological variables used and unused in analyses are in columns prior to arthropod species.
(XLSX)

S3 File A file that provides species lists indicating plant biome and season collected. (XLSX)
