# Supplementary figures and images for: Ground-Dwelling Arthropod Communities of a Sky Island Mountain Range in Southeastern Arizona, USA: Obtaining a Baseline for Assessing the Effects of Climate Change
Source: PLoS One. 2015 Sep 2;10(9):e0135210. doi: 10.1371/journal.pone.0135210 (PMC4558002; doi:10.1371/journal.pone.0135210)

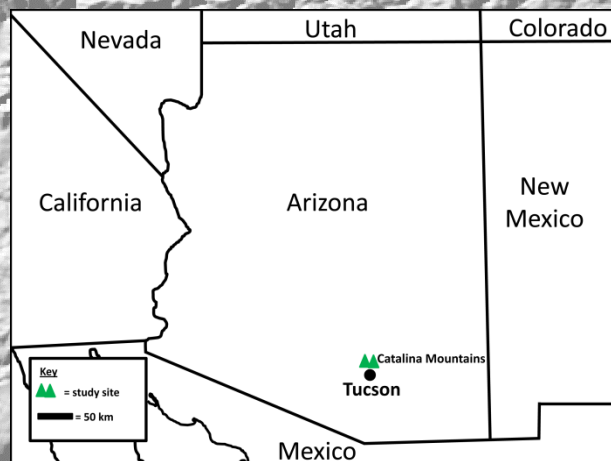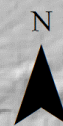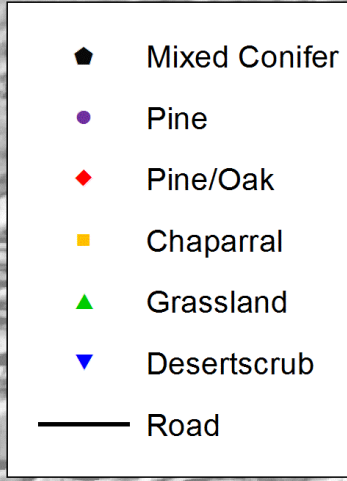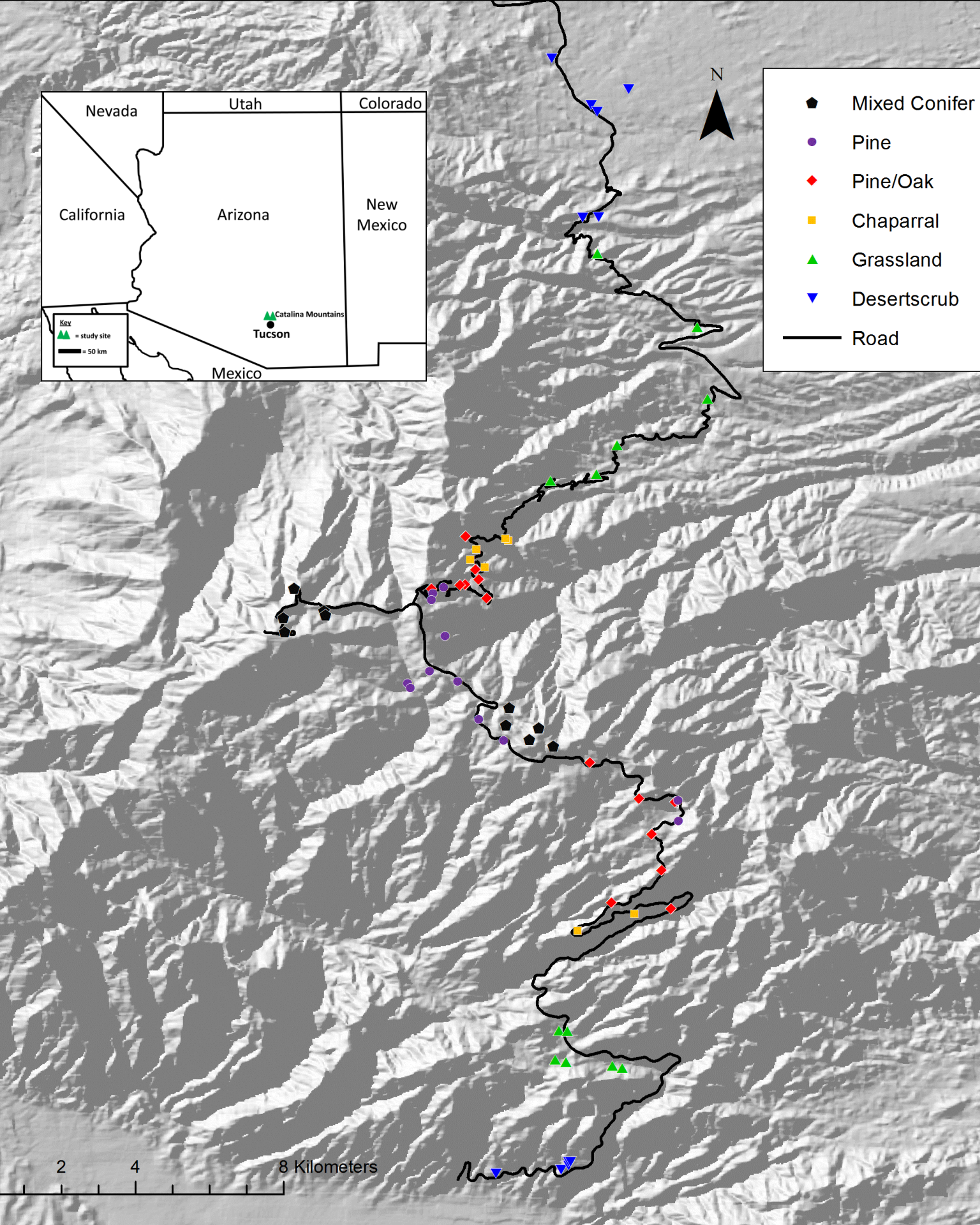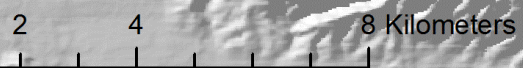

Supplement: S1 File — Color coding of sites according to plant biomes is consistent with Figs 2 and 3. (PDF) [file pone.0135210.s001.pdf]
